# Machine learning models in heart failure with mildly reduced ejection fraction patients

**DOI:** 10.3389/fcvm.2022.1042139

**Published:** 2022-11-30

**Authors:** Hengli Zhao, Peixin Li, Guoheng Zhong, Kaiji Xie, Haobin Zhou, Yunshan Ning, Dingli Xu, Qingchun Zeng

**Affiliations:** ^1^State Key Laboratory of Organ Failure Research, Department of Cardiology, Nanfang Hospital, Southern Medical University, Guangzhou, China; ^2^Guangdong Provincial Key Laboratory of Shock and Microcirculation, Southern Medical University, Guangzhou, China; ^3^Bioland Laboratory (Guangzhou Regenerative Medicine and Health Guangdong Laboratory), Guangzhou, China; ^4^School of Laboratory Medicine and Biotechnology, Southern Medical University, Guangzhou, China

**Keywords:** heart failure, machine learning (ML), heart failure with mildly reduced ejection fraction, random forest (RF), LASSO Cox regression analysis

## Abstract

**Objective:**

Heart failure with mildly reduced ejection fraction (HFmrEF) has been recently recognized as a unique phenotype of heart failure (HF) in current practical guideline. However, risk stratification models for mortality and HF re-hospitalization are still lacking. This study aimed to develop and validate a novel machine learning (ML)-derived model to predict the risk of mortality and re-hospitalization for HFmrEF patients.

**Methods:**

We assessed the risks of mortality and HF re-hospitalization in HFmrEF (45–49%) patients enrolled in the TOPCAT trial. Eight ML-based models were constructed, including 72 candidate variables. The Harrell concordance index (C-index) and DeLong test were used to assess discrimination and the improvement in discrimination between models, respectively. Calibration of the HF risk prediction model was plotted to obtain bias-corrected estimates of predicted versus observed values.

**Results:**

Least absolute shrinkage and selection operator (LASSO) Cox regression was the best-performing model for 1- and 6-year mortality, with a highest C-indices at 0.83 (95% CI: 0.68–0.94) over a maximum of 6 years of follow-up and 0.77 (95% CI: 0.64–0.89) for the 1-year follow-up. The random forest (RF) showed the best discrimination for HF re-hospitalization, scoring 0.80 (95% CI: 0.66–0.94) and 0.85 (95% CI: 0.71–0.99) at the 6- and 1-year follow-ups, respectively. For risk assessment analysis, Kansas City Cardiomyopathy Questionnaire (KCCQ) subscale scores were the most important predictor of readmission outcome in the HFmrEF patients.

**Conclusion:**

ML-based models outperformed traditional models at predicting mortality and re-hospitalization in patients with HFmrEF. The results of the risk assessment showed that KCCQ score should be paid increasing attention to in the management of HFmrEF patients.

## Graphical Abstract

In this study, we first selected heart failure patients whose LVEF was 45–49%. We then developed eight machine learning-based prediction models for the outcomes of all-cause mortality and HF rehospitalization. We selected the best-performing prediction model for different outcomes and then further demonstrated the risk factors involved. 
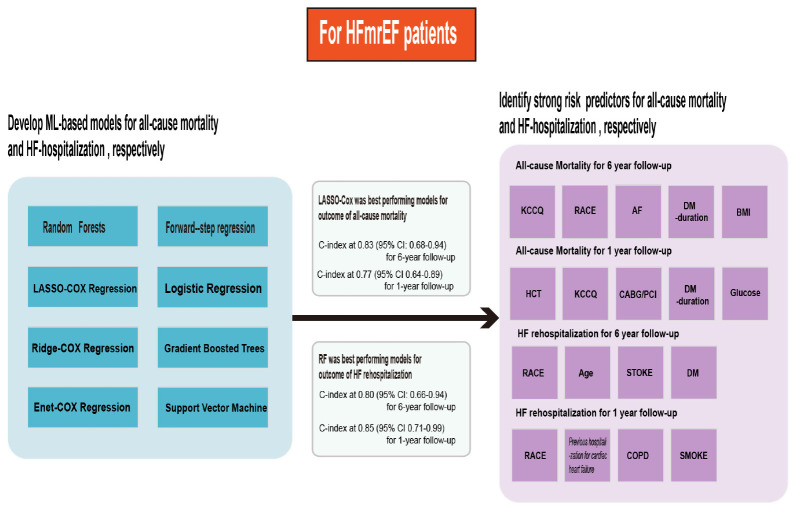

## Introduction

Heart failure (HF), a major public health concern, has affected an estimated 20 million patients globally and has become one of the leading causes of hospitalization in adults >65 years, making it a substantial threat to human health.

The 2021 ESC guidelines for chronic HF categorize patients into three subgroups based on whether their left ventricular ejection fraction (LVEF) is reduced (HFrEF, EF ≤40%), mildly reduced (HFmrEF; EF 41–49%), or preserved (HFpEF; EF ≥50%). Among these subgroups, HFmrEF has recently attracted increasing attention (1). Data from the ESC Heart Failure Long-Term Registry showed that in the outcome of all-cause mortality, there was no significant difference in all-cause mortality between HFmrEF and HFrEF or HFpEF, while the mortality rate was markedly higher among HFrEF patients than among HFpEF patients (2). Regarding outcomes of 1-year death and hospitalization incidences, HFmrEF and HFpEF patients showed lower rates than HFrEF patients. Indeed, the clinical characteristics, risk prediction and therapeutic strategy of HFmrEF are still obscure. Accurately predicting outcomes such as mortality and rehospitalization in HF is critically important to patients, their clinicians and healthcare systems, but it has proven to be a difficult task because the outcomes are affected by many risk factors.

Machine learning (ML) is a scientific discipline that focuses on how computers learn from data to improve predictive performance and generalization of models by considering higher-dimensional and possibly non-linear effects of variables, incorporating more variables (3). It has been extensively utilized in the cardiovascular field of diagnosis, image analysis and risk assessment (4). Compared with conventional statistical models, it has the ability to automatically learn from large datasets with a labeled output or outcome to conduct predictive analytics, allowing the user to glean knowledge from past data and apply it to future predictions. Recent evidence indicates that ML-based HF risk models that include clinical, laboratory, and biomarker data have demonstrated superior performance over traditional HF risk models but have been verified only in HFrEF and HFpEF populations. Therefore, predictive models for HF with HFrEF or HFpEF are available, but risk assessments of death and hospitalization in patients with HFmrEF are still limited.

## Materials and methods

### Study population

The design, enrollment criteria, and participant characteristics of the TOPCAT trial have been described previously. Briefly, it is a multicenter, randomized, double-blind, placebo-controlled trial of aldosterone antagonist therapy (NCT00094302), which includes 3,445 adult patients with symptoms of HF and documented LVEF ≥45%, aged 50 years or older (5). In the present analysis, we selected 519 patients whose LVEF was 45–49%, the data collected included all baseline demographics, clinical data, laboratory results, electrocardiography and Kansas City Cardiomyopathy Questionnaire (KCCQ) scores. A detailed description is provided in the supplement and a list of markers is shown in Supplementary Table 1.

### Outcomes of interest

The outcomes of interest in this study were all-cause mortality and HF hospitalization through 1 year and the entire follow-up (up to 6 years per subject). All-cause mortality was defined as death from any cause, and hospitalization for HF was defined as sudden presentation to an acute care facility with aggravated HF requiring overnight hospitalization.

### Candidate variables

In the present analysis, 87 candidate variables were considered, including all baseline demographics, clinical data, laboratory results, electrocardiography, and KCCQ scores. Some categorical candidate variables were harmonized and merged to facilitate analysis. A total of 72 predictor variables were included after excluding 6 covariates for a >20% missing rate and 8 for merged values, and another EF value was used as a screening condition and was not considered a variable (Supplementary Table 1).

### Model development and evaluation

**FIGURE 1:**
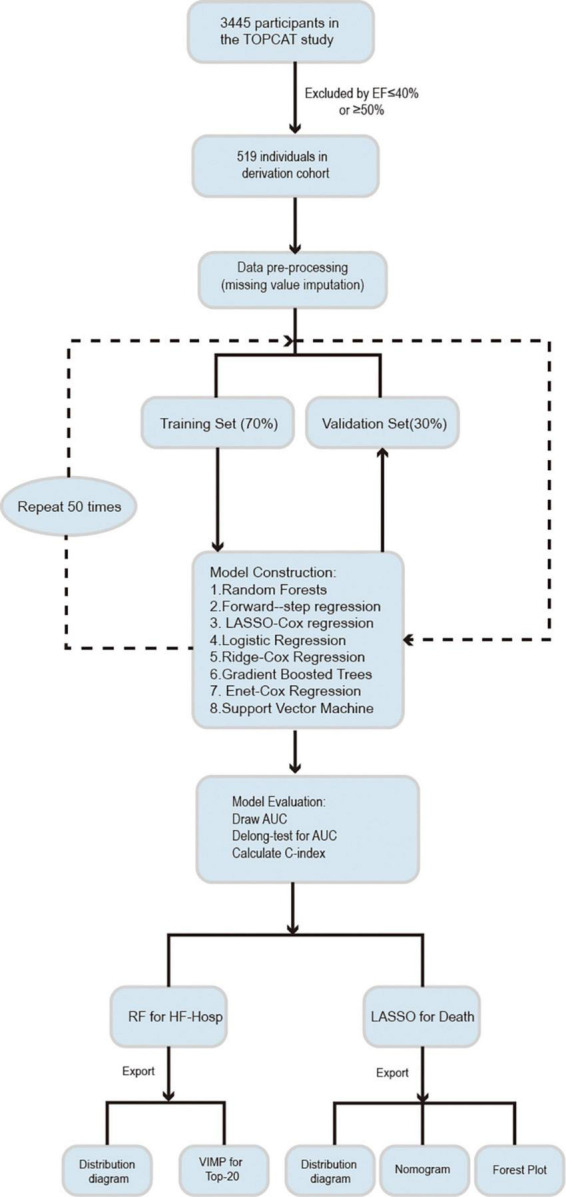
Analysis overview for identifying best performing risk prediction model.

The study population was randomly split into training (70%) and validation datasets (30%). Data imputation was performed on each dataset separately by using the missForest approach, which can cope with different types of variables, especially for multivariate data consisting of continuous and categorical variables (6). Different methods were used to model and optimize the training datasets to reduce the prediction error. These models were then checked on validation subsets to test the models’ performance and determine the best predictors. All of the steps were repeated 50 times. The analytical procedures followed in this study are shown in Figure 1.

### Machine learning-based methods

Heart failure prediction models were developed by incorporating the 72 variables identified previously, yielding the following eight candidate ML-based and conventional Cox regression algorithms for assessing the risk of mortality and HF hospitalization through 1 and 6 years of follow-up:

Random forest (RF);
Forward stepwise Cox regression;
Least absolute shrinkage and selection operator (LASSO) Cox regression;
Logistic regression;
Ridge Cox regression;
Gradient boosted trees;
Elastic net Cox regression;
Support vector machine (SVM).

Analyses were performed using R version 4.0.4 (R Foundation for Statistical Computing, Vienna, Austria). Various R packages were used to conduct this analysis. The package missForest (6) was used for imputation, randomForest (7) was used for RF, glmnet (8) was used for LASSO, ridge and elastic-net Cox regression, and the package gbm (9) was used for gradient boosted trees. e1071 (10) software was used for the SVM.

### Model evaluation

The discriminatory performance of each model against the validation dataset was calculated using the Harrell concordance index (C-index) (11) or the area under the receiver operating characteristic (ROC) curve (AUC). The DeLong test was used to assess discrimination between models (12). Calibration of the HF risk prediction model was plotted to obtain bias-corrected (overfitting-corrected) estimates of predicted versus observed values based on subsetting the predictions into intervals. The prediction distribution of the models was plotted based on the order of the predicted risk for each patient.

### Sensitivity analyses

Sensitivity analysis was computed for all patients from the TOPCAT study whose LVEF was 45–49%. These different models were developed for this population to predict all-cause mortality and HF hospitalization and were followed throughout the study period (13). The importance of each variable was calculated, and the incremental improvement in each variable was assessed over 50 cycles of simulation. In addition, 1-year all-cause mortality and HF hospitalization predictions were evaluated to see how the model’s performance changed over a relatively short follow-up period.

## Results

### Study cohort and participant baseline characteristics

A total of 519 patients with LVEF values ranging from 45 to 49% were included (Table 1), of whom 63.5% were male and 91.3% were white, with a mean age of 66.1 years and a median body mass index (BMI) of 31.4 kg/m^2^. Over a 6-year follow-up, a total of 97 patients died, accounting for 18.6% of the total number of participants, and 59 patients (11.3%) were hospitalized for worsening HF. During the first year of follow-up, the incidence of all-cause mortality and HF hospitalization was 5.1% (31) and 4.6% (24), respectively. Among the imputation cohort, some candidate variables, for example, glucose, alkaline phosphatase (ALP), hematocrit, waist circumference, and physical limitation, had missing values. After processing them, they exhibited close agreement with the original data, which showed that the method we chose was reliable.

**TABLE 1:** Baseline characteristics of patients (*N* = 519).

	Original cohort	Imputation cohort
Male	330 (63.5%)	330 (63.5%)
Age	66.1 (9.23)	66.1 (9.23)
White patients	474 (91.3%)	474 (91.3%)
Heart rate, bpm	69.7 (13.39)	69.7 (13.39)
SBP	127.9 (13.80)	127.9 (13.80)
DBP	77.5 (9.72)	77.5 (9.72)
BMI	31.4 (7.01)	31.4 (7.01)
Serum potassium	4.26 (0.47)	4.26 (0.47)
Serum calcium	102.74 (6.32)	102.77 (6.19)*
Serum sodium	141.40 (4.32)	141.40 (4.32)
Creatinine	1.11 (0.30)	1.10 (0.30)*
HCT	40.85 (5.54)	40.85 (5.52)*
WBC	7.05 (2.05)	7.05 (2.05)
Waist Circumference	104.84 (16.67)	104.88 (16.66)*
GFR	69.67 (19.91)	69.67 (19.91)
**NYHA_CLASS**		
I and II	335 (64.5%)	335 (64.5%)
III and IV	184 (35.4%)	184 (35.5%)
Current smoker	81 (15.6%)	81 (15.6%)
Ever-smoker	206 (39.7%)	206 (39.7%)
Hemoglobin, g/dl	13.58 (1.74)	13.58 (1.74)
Glucose	112.74 (41.37)	112.83 (41.30)*
ALP	105.24 (60.71)	105.42 (59.83)*
QRS duration, ms	102.13 (31.14)	102.13 (31.14)
Cooking salt score	4.75 (3.62)	4.75 (3.62)
**KCCQ scores**		
Physical limitation	56.38 (22.77)	56.39 (22.68)*
Symptom stability score	51.78 (24.13)	51.78 (24.13)
Symptom frequency score	57.95 (24.30)	57.95 (24.30)
Symptom burden score	59.59 (23.78)	59.59 (23.78)
Total symptom score	58.77 (23.06)	58.77 (23.06)
Self-efficacy score	65.99 (24.72)	65.99 (24.72)
Quality of life score	49.78 (22.86)	49.78 (22.86)
Overall summary score	54.68 (21.13)	54.68 (21.13)
Clinical summary score	57.60 (21.02)	57.60 (21.02)

ALP, alkaline phosphatase; BMI, body mass index; DBP, diastolic blood pressure; GFR, glomerular filtration rate; HCT, hematocrit; KCCQ, Kansas City Cardiomyopathy Questionnaire; NYHA, new york heart association; SBP, systolic blood pressure; WBC, white blood count.

*Indicates imputation cohort is different from the original cohort.

### Machine learning for prediction of heart failure mortality outcome

The 72 predictor covariates incorporated into the risk prediction models included demographics, clinical history, vital signs, social history, laboratory, and electrocardiographic parameters (Supplementary Table 1).

**FIGURE 2:**
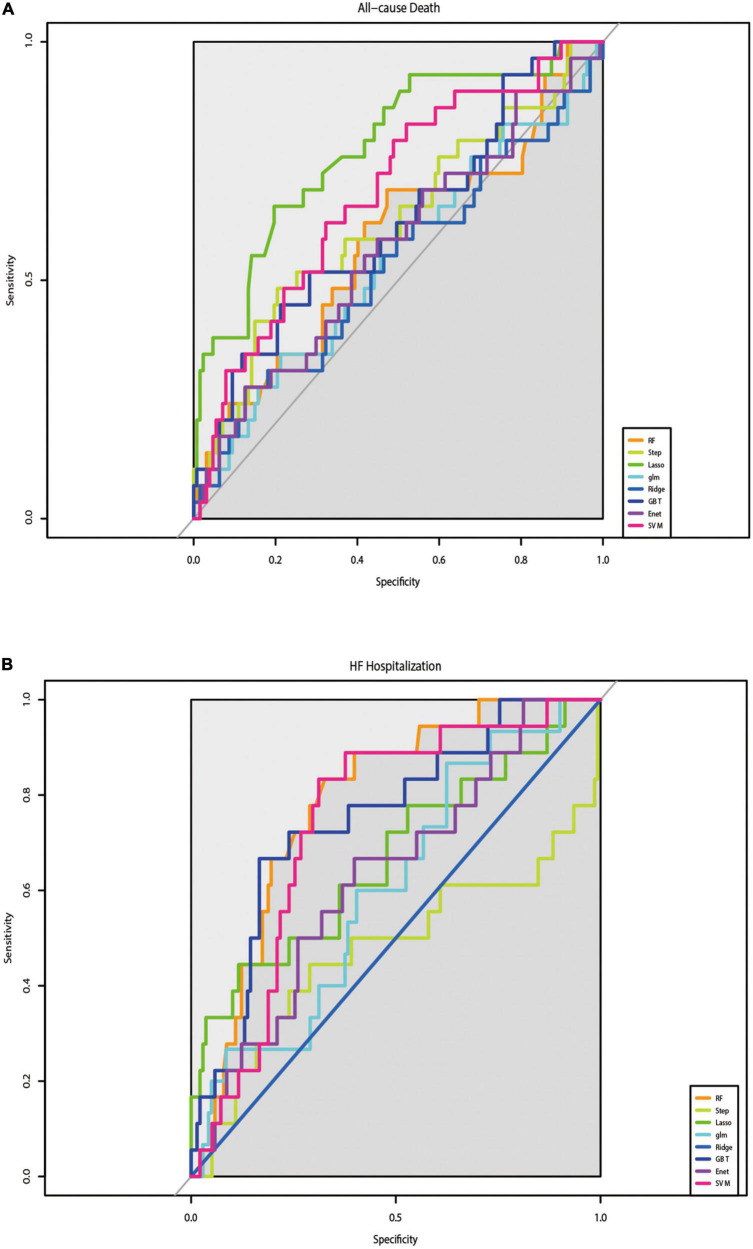
Results of the discrimination for all-cause mortality and HF-Hospitalization in ROC curves. **(A)** The performance of eight prediction models for all-cause mortality was assessed by ROC curves. **(B)** The performance of eight prediction models for re-hospitalize was assessed by ROC curves.

The C-indices and C-statistic for ML-based HFmrEF risk prediction models are displayed in Table 2. The results of eight prediction models for all-cause mortality showed that LASSO Cox regression performed the best at both the 1- and 6-year follow-ups. Compared with the other seven models, LASSO Cox regression had the highest overall C-statistic, at 0.78 over 6 years and 0.75 for 1 year. The C-indices for LASSO regression were also the highest, at 0.83 [95% confidence interval (CI): 0.68–0.94] and 0.77 (95% CI: 0.64–0.89) at the 6- and 1-year follow-ups, respectively. This was in contrast to the ridge regression model; across both short and long follow-ups, the ridge Cox regression model had the lowest C-index [1 year: 0.52 (95% CI: 0.38–0.65); 6 years: 0.51 (95% CI: 0.38–0.63)]. Figure 2A shows the ability of the models to discriminate groups by mortality using ROC curves.

**TABLE 2:** Discrimination of the models for mortality.

	Death-6 year			Death-1 year		
		
	C-index	AUC	DeLong test	C-index	AUC	DeLong test
RF	0.56 (0.42–0.69)	0.58	0.0297*	0.67 (0.54–0.80)	0.68	0.6682
Step-forward	0.68 (0.59–0.82)	0.62	0.0126*	0.50 (0.36–0.64)	0.49	0.0011*
Lasso	0.83 (0.66–0.94)	0.78	1	0.77 (0.64–0.89)	0.75	1
Logistic	0.55 (0.41–0.69)	0.54	0.0051*	0.53 (0.40–0.66)	0.46	0.0095*
Ridge	0.52 (0.38–0.65)	0.53	0.0057*	0.51 (0.38–0.63)	0.52	0.0900
GBT	0.61 (0.45–0.73)	0.62	0.0530	0.76 (0.62–0.89)	0.77	0.9209
Elastic-net	0.54 (0.44–0.71)	0.55	0.0160*	0.54 (0.41–0.67)	0.54	0.2378
SVM	0.58 (0.47–0.69)	0.58	0.0036*	0.64 (0.54–0.75)	0.62	0.4207

GBT, gradient boosted trees; RF, random forest; SVM, support vector machine; AUC, area under the curve.

*Indicates *p* < 0.05.

### Machine learning for prediction of heart failure hospitalization outcome

Table 3 shows the results of the C-indices and C-statistic for eight prediction models of HF hospitalization. Of the eight models, RF showed the best discrimination, with the highest overall C-statistic of 0.80 over a maximum of 6 years of follow-up and 0.85 for 1 year. The C-indices for RF were also the highest, at 0.80 (95% CI: 0.66–0.94) and 0.85 (95% CI: 0.71–0.99) at the 6- and 1-year follow-ups, respectively. The DeLong test showed that the RF model was different from the step-forward and ridge regression models, especially the latter (*p*-value = 0.0017, <0.0001, respectively). The performance of the models in discriminating HF hospitalization was assessed by ROC curves (Figure 2B).

**TABLE 3:** Discrimination of the models for HF hospitalization.

	HF-hospitalization-6 year			HF-hospitalization-1 year		
		
	C-index	AUC	DeLong test	C-index	AUC	DeLong test
RF	0.80 (0.66–0.94)	0.78	1	0.85 (0.71–0.99)	0.84	1
Step-forward	0.56 (0.43–0.70)	0.48	0.0017*	0.67 (0.53–0.81)	0.67	0.0503
Lasso	0.72 (0.59–0.86)	0.67	0.2429	0.49 (0.36–0.62)	0.47	0.0016
Logistic	0.62 (0.48–0.76)	0.60	0.0437*	0.43 (0.33–0.54)	0.58	0.1632
Ridge	0.50 (0.34–0.66)	0.50	<0.0001*	0.63 (0.51–0.75)	0.39	0.0037
GBT	0.77 (0.63–0.91)	0.75	0.3605	0.81 (0.67–0.94)	0.81	0.6428
Elastic-net	0.65 (0.51–0.78)	0.63	0.0048*	0.61 (0.50–0.73)	0.60	0.0130
SVM	0.78 (0.64–0.93)	0.74	0.3488	0.70 (0.59–0.82)	0.40	0.3292

GBT, gradient boosted trees; RF, random forest; SVM, support vector machine; AUC, area under the curve.

*Indicates *p* < 0.05.

### Characteristic variables of mortality

**FIGURE 3:**
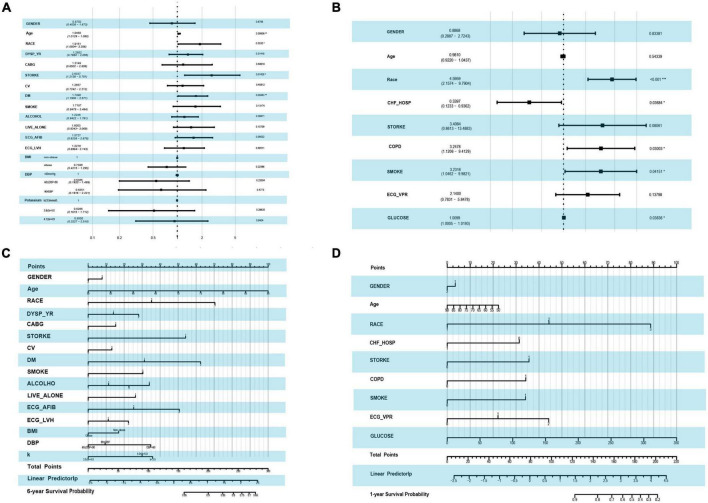
Forest plot by using the multi-variable COX regression and the risk score nomogram. **(A)** Forest plot of variables selected by LASSO COX regression in the end point event of all-cause mortality at 6-year follow-up. **(B)** Forest plot of variables selected by LASSO in the end point event of all-cause mortality at 1-year follow-up. **(C)** Nomogram for predicting 6-year all-cause mortality based on variables selected by LASSO COX regression. **(D)** Nomogram for predicting 1-year all-cause mortality based on variables selected by LASSO COX regression.

For the outcome of mortality, LASSO regression showed the best performance. To improve clinical usability, we further constructed a model made of the variables filtered by LASSO regression. The forest plot of the variables found by multivariate Cox regression is shown in Figures 3A,B. During the 6-year follow-up, 16 covariates were selected by LASSO, and only four variables (age, race, stroke, and DM) played significant roles in the prediction models (*p*-values = 0.01, 0.03, 0.01, and 0.00, respectively) (Figure 3A). Figure 3B shows the nine independent variables selected by LASSO over 1 year of follow-up. Among the nine variables, race, previous hospitalization for cardiac heart failure (CHF-HOSP), chronic obstructive pulmonary disease (COPD), smoking, and blood glucose showed a significant influence on the short-term mortality of HFmrEF patients.

A risk score for 1- and 6-year mortality was created using a nomogram (Figures 3C,D). Scores for the 6-year follow-up, ranging from 0 to 300, were assigned points as follows: for age, the points went from 0 to 100, with higher scores for older age. For race, white had a score of 0, black 35 and other races 70 points. Patients with HFmrEF without diabetes, with diabetes, and with diabetes-related microvascular complications showed increasing risk scores of 0, 35, and 70 points, respectively. Figure 3D shows the risk scores at the 1-year follow-up. Among the nine variables, the race score ranged from 0 to 89, CHF-HOSP ranged from 0 to 31 points, COPD added a risk score of 34 points and its absence 0, and the glucose score was positively correlated with risk points at the 6-year follow-up. The calibration of the LASSO-based model is plotted in Supplementary Figure 1.

### Characteristic variables of heart failure hospitalization

**FIGURE 4:**
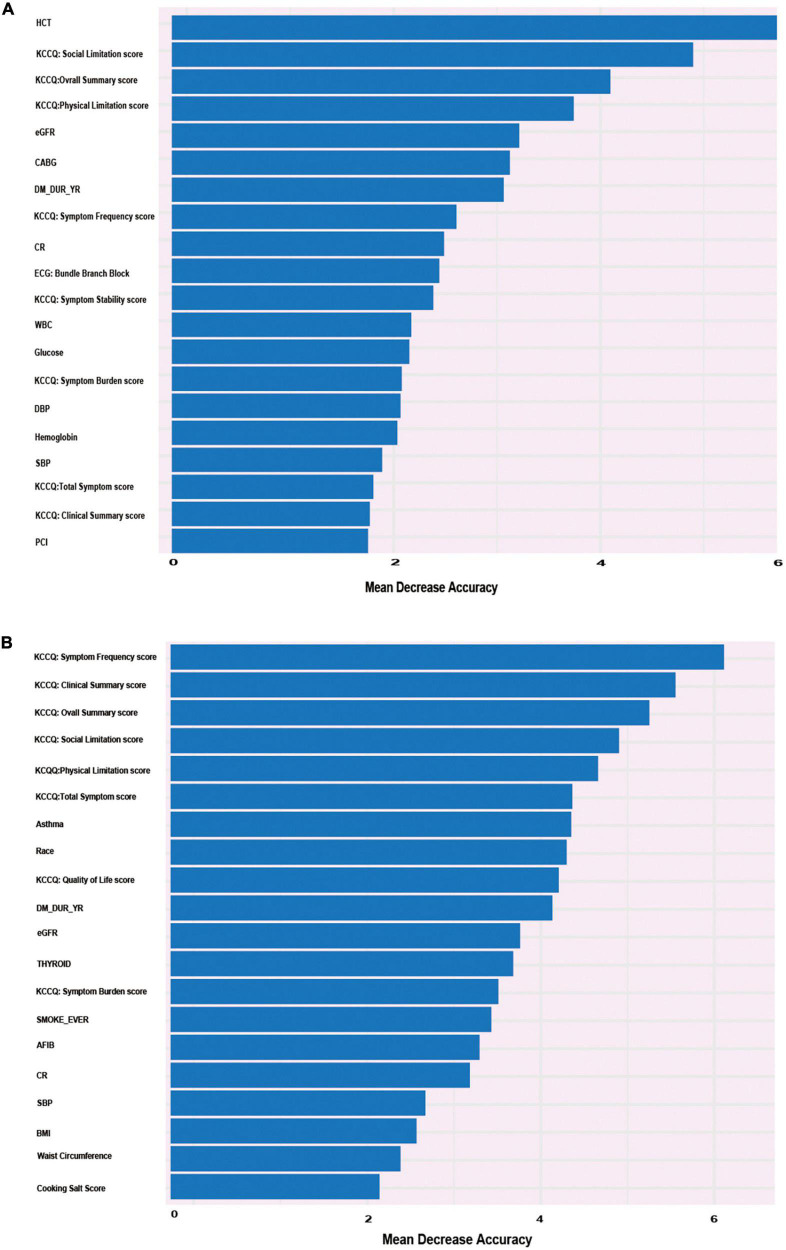
Alluvial plot of the 20 most important parameters for HF re-hospitalization risk prediction identified by the VIMP metric in the RF model in the derivation cohort. **(A)** Twenty most important parameters for predicting the risk of re-hospitalization for heart failure at 6-year follow-up identified by VIMP method. **(B)** Twenty most important parameters for predicting the risk of re-hospitalization for heart failure at 1-year follow-up identified by VIMP method.

The RF model showed the best performance at predicting the outcome of HF hospitalization. RF addressed each feature in the order of mean decrease accuracy to rank the importance of its variables. To further clarify the important variables, we graphed the top 20 covariates identified by the variable importance (VIMP) metric (Figure 4). Values of mean decrease accuracy are shown in Supplementary Tables 2, 3.

Figure 4A shows that KCCQ scores, including symptom frequency, clinical summary, overall summary, social limitation, physical limitation, and total symptoms, all exhibited a major influence on long-term HF hospitalization. Asthma, race, and BMI also played important roles in the prediction model. Compared with the long-term follow-up characteristics, which were closely related to quality of life, variables that predicted short-term HF hospitalization were more correlated with previous clinical history and clinical laboratory results, such as hematocrit, estimated glomerular filtration rate (eGFR), creatinine (CR), coronary artery bypass graft (CABG) surgery, and percutaneous coronary intervention (PCI). The candidate variables are ranked by importance in Figure 4B. The calibration of the RF-based model is plotted in Supplementary Figure 2.

### Distribution of outcomes

**FIGURE 5:**
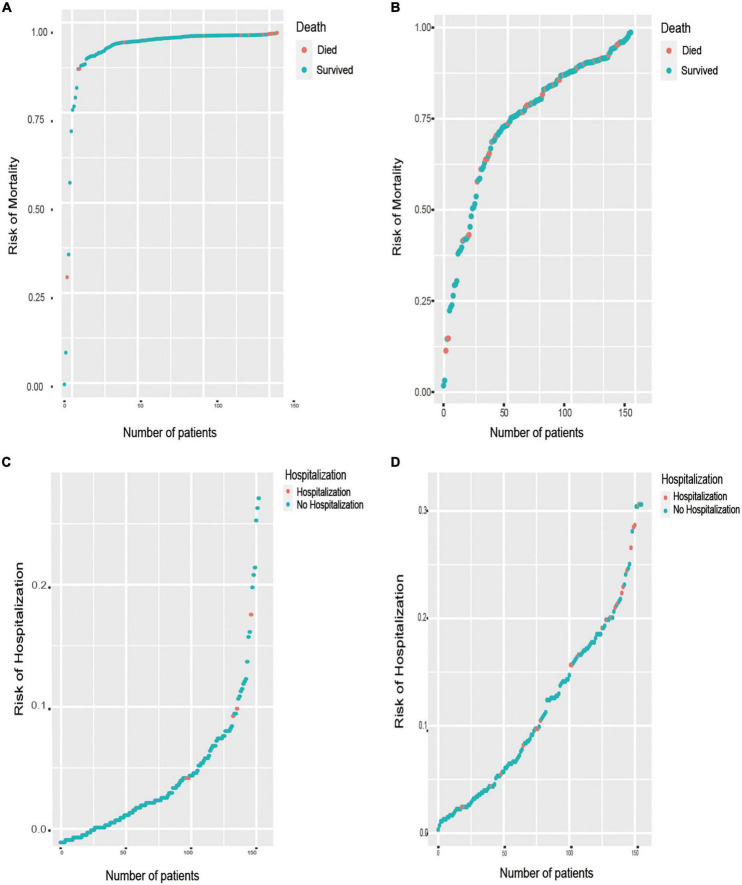
Prediction distributions of patients with HFmrEF in the outcomes of all-cause mortality and HF hospitalization. **(A)** The distribution of all-cause mortality at the 1-year follow-ups, which there is a positive correlation between the number of patient deaths and the risk of mortality. **(B)** The distribution of all-cause mortality at the 6-year follow-ups. **(C)** The distribution of re-hospitalization at the 1-year follow-ups, which there is a positive correlation between the number of patients’ hospitalization and the risk of hospitalization. **(D)** The distribution of re-hospitalization at the 6-year follow-ups.

Figure 5 shows the predicted distribution of the best performance models, which were sorted by risk. These models were LASSO Cox regression for the outcome of all-cause death and RF for hospitalization with HF exacerbation. The prediction models with positive clustering of patients who died or were hospitalized with HF aggravation (Figure 5) indicated that the models accurately stratified patients at risk of death and hospitalization. Figures 5A,B show the distribution of all-cause mortality at the 1- and 6-year follow-ups, respectively. Figure 5C shows the distribution of HF hospitalizations at the 1-year follow-up, and Figure 5D shows the distribution at the 6-year follow-up.

## Discussion

Heart failure with an LVEF of 40–49% was first established as a category in 2013. Later, in 2016, the ESC defined HF with an LVEF range of 40–49% as a new subtype of HF: heart failure with mildly reduced ejection fraction (HFmrEF), which was redefined to HF with a mildly reduced (41–49%) ejection fraction in the 2021 ESC guidelines (1, 14). Compared with HFpEF and HFrEF, HFmrEF is less well studied. The pharmacological treatment for HFmrEF has been updated in the 2021 ESC guidelines, which propose that diuretics, angiotensin-converting enzyme (ACE) inhibitors, angiotensin receptor blockers (ARBs), and beta blockers may be considered for this category to reduce the risks of HF hospitalization and death. But the issue of whether these patients represent a distinct HF subtype that may benefit from therapies salutary for patients with HFrEF requires further study.

Accurately predicting prognosis plays an important role in choosing a therapeutic regimen and improving the outcome of HF. In this cohort of 519 individuals in the TOPCAT trial with LVEF ranging from 41 to 49%, we developed and validated eight alternative risk models for the prediction of HF hospitalization and all-cause mortality. Our model includes abundant suits of clinical risk factors that are measurable and accessible in history taking. The RF model performed the best, with good validation and excellent accuracy and calibration for rehospitalization, and a LASSO regression model was the best model for mortality prediction.

In both prediction models, patients’ physical conditions, as evaluated and quantified by the KCCQ scores, were the strongest predictors of both death toll and HF readmission over a 6-year follow-up. When combined with NT-proBNP, KCCQ could serve as an optional tool for quick and convenient risk fractionation (15–17). In our models, KCCQ accounted for a large amount of mortality and HF readmission prediction because it represents a status health quality of life and could be influenced by many factors, such as gender, race, non-cardiovascular and cardiovascular comorbidities. In recent years, with the rapid development of the internet, smartphones, and wearable health devices, KCCQ can be obtained instantaneously for physicians working in telehealth (18). Therefore, the KCCQ has the advantage of a quick overview of patients’ HF risk for physicians in remote areas or clinics. The KCCQ also record clinically meaningful changes over time, making it promising to support joint decision-making and more efficient medical interventions to quickly identify patients at higher risk stratification of mortality and readmission.

Based on our results, AF seems to confer both short- and long-term risk of all-cause death and cardiovascular rehospitalization. In research carried out by David M. Kaye, both HFmrEF and HFpEF patients with AF had remarkably increased pulmonary capillary wedge pressure, decreased cardiac index and reduced left ventricular work index. At similar levels of systemic circulation workload, AF patients fail to adapt their oxygen consumption to the increase in workload, which is accompanied by an irreversibly impaired cardiac index and ventricular working index (19). A cohort study in the ESC Heart Failure Long-Term Registry found that AF increased with increasing LVEF, accounting for poor cardiovascular outcomes only in HFpEF and HFmrEF patients and not in HFrEF patients (20). In our ML-based modeling results, AF is also one of the top predictors of all-cause mortality. However, the current guidelines suggest that patients with HFmrEF are less likely to suffer from AF and non-cardiac comorbidities. Therefore, the relationship between the occurrence of AF and the prognosis of HFmrEF warrants further study and exploration.

High BMI is proven a risk factor for HF, patients with a normal or low BMI have a higher mortality and readmission rate than those with a relatively high BMI. The phenomenon is termed the “obesity paradox” (21–25). This also existed according to an investigation of HFmrEF patients (26). In our ML-based models, patients with high BMI had lower scores than those with low BMI. To the best of our knowledge, a high percentage of body fat mass indicates good nutrition situations, and this is probably relevant to a lower risk of short-term relapse of cardiac events in HFmrEF patients. Moreover, it is also considered that the obesity paradox may be attributed to the intrinsic limitations of BMI as an index of obesity. Other body mass measures, such as body fat distribution, body fat rate and fat-free mass, are probably more accurate for examining the relationship of body composition with health outcomes. For instance, Chandramouli et al. recently reported that the obesity paradox is manifested only when BMI is used as a weight parameter. When the waist-to-height ratio (WHtR) is used, the opposite association emerges (26). Therefore, further studies are needed to develop metrics for better analysis of body composition, better estimation of various obesity phenotypes and better prediction of mortality and rehospitalization in HF.

Another risk factor, eGFR was significant in our predicting model for mortality and readmission in cohort of HFmrEF. Patients with chronic HF are vulnerable to renal impairment (RI), and conversely, impaired renal function is associated with a higher mortality risk in HF patients. Research examining the relationship between all subtypes of HF and the prognostic impact of chronic kidney disease shows that in HFpEF patients, although the incident rate of CKD is higher, CKD is less important with a weaker correlation with all-cause death, have a less risk score compared with conventional risk markers, and exerts insufficient differentiation for prediction of mortality (27). These findings are in line with our results. In the cohort of HFmrEF in the TOPCAT trial, eGFR was a more powerful predictor of mortality in patients with HFmrEF than in those with HFpEF. We speculate that morbidity of CKD may give rise to sympathetic and neurohormonal activation and cause further deterioration of HF. This was also believed to be associated with other underlying diseases that impair renal function such as hypertension and diabetes which are prevalent among HFmrEF patients. This link was further evidenced by the Meta-Analysis Global Group in Chronic Heart Failure (MAGGIC) meta-analysis, which showed a lower mortality rate and lower association between CKD and death in patients with HFpEF than in HFmrEF (28).

Consistent with the 6-year findings, LASSO regression and the RF method showed the best predictive performance for 1-year mortality and readmission, respectively. Interestingly, unlike the top 20 risk factors screened by the RF model in the 6-year rehospitalization prediction, hematocrit (HCT) was proposed as one of the most important risk factors. Although association between HCT and incident HF has not been well established, several follow-up studies have elucidated that higher levels of HCT were associated with an increased risk of developing HF and coronary events (29–32). Additionally, Gagnon et al. proposed that both low and high HCT levels were positively associated with the occurrence of cardiovascular events (33). All of the above-mentioned findings suggest that the usage of hemoglobin and HCT for the estimation of plasma volume may represent a useful tool in the field of HF. Recently, estimated plasma volume status (ePVS), a relatively simple and non-invasive plasma volume estimation based on hemoglobin and hematocrit, was prompted to be a better predictor of both post-discharge and bedside clinical assessments (34). Kobayashi et al. and Grodin proposed that ePVS was associated with systemic congestion and deterioration of HF, regardless of other influencing factors (32, 35). Consequently, it could be a useful congestion index in patients with HF, in line with our findings in HFmrEF patients. Hemodynamic congestion develops and progresses slowly but eventually gives rise to symptomatic congestion and consequently urgent hospitalization. Accordingly, HCT may represent a convenient clinical indicator in patients with HFmrEF. This also suggests that ePVS might be an additional phenotypic characteristic considered for clinical study and for tailoring personalized therapies for HF patients.

DM has been recognized as an independent risk factor for the development of HF. Previous study conducted by Bhambhani et al. have reported that diabetes mellitus could predict the incident of HFmrEF, and this finding could be further confirmed in our study (36). In this study, we found DM was one of the strongest predictors of both the primary and mortality endpoints in the HFmrEF patients. And DM patients with HF treated with sodium–glucose co-transporter 2 inhibitors (SGLT2i) have shown impressive protective effects (37). In addition, the importance of other predictors in the prediction of readmission and mortality of HFmrEF differed greatly, including BP, smoking, age and stroke for predicting death, and WBC, CR and Salt intake for predicting HF re-hospitalization. In this regard, ML improved the prediction accuracy, letting us find novel relationships that were not hypothesis driven and shed light on some ignored risk factors.

Our study also has certain limitations. First, the TOPCAT trial was conducted between 2006 and 2012. Missing values of biomarkers such as circulating natriuretic peptides and high-sensitivity troponin affected our analysis and were not available to assess dynamic risk prediction scores. And due to the time period of the TOPCAT study, patients with HFmEF were not treated with SGLT-2 antagonists, which could alter the risk profile of these patients and potentially affected the model outputs. Second, we enrolled 519 patients with LVEF ranged from 41 to 49%. Unfortunately, the TOPCAT trial excluded the population with an LVEF greater than 45%. Therefore, we did not include adult patients with symptoms of HF and documented LVEF <45%. Third, given that our research is a *post hoc* analysis of the TOPCAT trial, and the TOPCAT study population was predominantly white males, therefore, our predicting models may not perform as well to the general population. Therefore, further validation of the role of ML in phenomenological mapping and sex-specific classification criteria is needed in a wide range of HFmrEF clinical data.

## Conclusion

Machine learning-based models outperformed traditional models at predicting mortality and re-hospitalization in patients with HFmrEF. The results of the risk assessment showed that KCCQ score should be paid increasing attention to in the management of HFmrEF patients.

## Data availability statement

The original contributions presented in this study are included in the article/Supplementary material, further inquiries can be directed to the corresponding authors.

## Author contributions

HeZ, PL, YN, DX, and QZ contributed to the design of the work. HeZ, PL, and GZ contributed to the analysis of the work. HeZ, PL, GZ, KX, and HaZ contributed to the interpretation of data. HeZ, PL, KX, YN, DX, and QZ wrote the original manuscript. HeZ, GZ, KX, HaZ, YN, DX, and QZ revised the manuscript for important intellectual content. HeZ, GZ, HaZ, YN, and QZ wrote the revised manuscript. KX, HaZ, YN, DX, and QZ approved the revised version to be published. HeZ, PL, GZ, KX, HaZ, YN, DX, and QZ agreed to be accountable for all aspects of the work. All authors contributed to the article and approved the submitted version.

## Abbreviations

HF: heart failure
ML: machine learning
LVEF: left ventricular ejection fraction
HFrEF: heart failure with reduced ejection fraction
HFmrEF: heart failure with mildly reduced ejection fraction
HFpEF: heart failure with preserved ejection fraction
KCCQ: Kansas City Cardiomyopathy Questionnaire
RF: random forest
LASSO: least absolute shrinkage and selection operator
C-index: Harrell concordance index
ROC: receiver operating characteristic
AUC: area under the receiver operating characteristic curve
BMI: body mass index
ALP: alkaline phosphatase
CHF-HOSP: hospitalization for cardiac heart failure
COPD: chronic obstructive pulmonary disease
VIMP: variable importance
eGFR: estimated glomerular filtration rate
CR: creatinine
CABG: coronary artery bypass graft
PCI: percutaneous coronary intervention
ACE: angiotensin-converting enzyme
ARBs: angiotensin receptor blockers
NT-proBNP: N-terminal pro-brain natriuretic peptide
WHtR: waist-to-height ratio
HCT: hematocrit
ePVS: estimated plasma volume status.
