# Supplementary material for: Machine learning models in heart failure with mildly reduced ejection fraction patients
Source: Front Cardiovasc Med. 2022 Nov 30;9:1042139. doi: 10.3389/fcvm.2022.1042139 (PMC9748556; doi:10.3389/fcvm.2022.1042139)
Supplement: Supplementary file 1 [file Data_Sheet_1.PDF]

Machine Learning Models in Heart Failure with Mildly Reduced Ejection Fraction patients

eMethods

Study population

The design, enrollment criteria, and participant characteristics of the TOPCAT trial have been described previously. Briefly, it is a multicenter, randomized, double-blind, placebo-controlled trial of aldosterone antagonist therapy (NCT00094302), which includes 3,445 adult patients with symptoms of HF and documented LVEF ≥ 45%, aged 50 years or older(1). The data collected included demographic and clinical data as well as data on quality of life and adherence to prescribed treatments(2). In this article, we selected 519 patients whose LVEF was 41-49%, and the research materials used in this article were obtained from the National Institutes of Heart, Lung, and Blood Institute's Biologic Specimen and Data Repository Information Coordinating Center via an approved proposal.

Outcomes of interest

The outcomes of interest in this study were all-cause mortality and HF hospitalization through 1 year and the entire follow-up (up to 6 years per subject). All-cause mortality was defined as death from any cause, and hospitalization for HF was defined as sudden presentation to an acute care facility with aggravated HF requiring overnight hospitalization.

Candidate variables

In the present analysis, 87 candidate variables were considered, including all baseline demographics, clinical data, laboratory results, electrocardiography and Kansas City Cardiomyopathy Questionnaire (KCCQ) scores. Some categorical candidate variables were harmonized and merged to facilitate analysis. A total of 72 predictor variables were included after excluding 6 covariates for a >20% missing rate and 8 for merged values, and another EF value was used as a screening condition and was not considered a variable (Supplementary Table 1).

Model development and evaluation

The study population was randomly split into training (70%) and validation datasets (30%). Data imputation was performed on each dataset separately by using the miss Forest approach, which can cope with different types of variables, especially for multivariate data consisting of continuous and categorical variables(3). Different methods were used to model and optimize the training datasets to reduce the prediction error. These models were then checked on validation subsets to test the models' performance and determine the best predictors. All of the steps mentioned above were repeated 50 times. The analytical procedures followed in this study are shown in Figure 1.

4.1 ML-based methods

HF prediction models were developed by incorporating the 72 variables identified previously, yielding the following eight candidate ML-based and conventional Cox regression algorithms for assessing the risk of mortality and HF hospitalization through 1 year and 6 years of follow-up:

Random forest (RF);

Forward stepwise Cox regression;

Least absolute shrinkage and selection operator (LASSO) Cox regression;

Logistic regression;

Ridge Cox regression;

Gradient boosted trees;

Elastic net Cox regression;
Support vector machine.
Analyses were performed using R version 4.0.4 (R Foundation for Statistical Computing, Vienna, Austria). Various R packages were used to conduct this analysis. The package missForest(3) was used for imputation, randomForest(4) was used for RF, glmnet(5) was used for LASSO, ridge and elastic-net Cox regression, and the package gbm(6) was used for gradient boosted trees. e1071(7) software was used for the support vector machine (SVM).

4.2 Variable selection

The variables selected were adjusted to optimize the number of covariates included in the HF risk prediction model. RF is a collection of classification and regression trees(8), using binaries for predictive variables to determine outcome predictions; it can be used not only to develop prediction models but also to select variables (9,10). Variable importance (VIMP) was used to identify which variables play a key role in prediction based on permutation selection by using prediction error attributable to the variable(11). Briefly, the higher the VIMP value of a variable is, the more important the role it plays in model prediction because permutation will reduce the accuracy of prediction(12). Forward stepwise selection evaluates the significance of each variable based on likelihood ratio tests for the coefficients using maximum likelihood estimation ($p < 0.05$) and adds the independent variables that improve the model the most sequentially(13,14). Compared with linear regression, ridge regression, LASSO regression and elastic net regression can solve the disadvantages of overfitting, but ridge regression easily leads to the distortion of regression results(15). Although LASSO regression can select a parsimonious, predictive subset of variables using more stringent thresholds to construct the model, it will cause the model to be too simple and not conform to reality, while elastic net regression can not only fulfill the purpose of ridge regression by selecting important features but also remove the features that have little influence on the dependent variable, thereby achieving good results, as in LASSO regression(16,17).

4.3 Model evaluation

The discriminatory performance of each model against the validation dataset was calculated using the Harrell concordance index (C-index)(18) or the area under the receiver operating characteristic (ROC) curve (AUC). A higher C-index and highest AUC indicate better predictive performance. Performance is reported as the mean and 95% CI after running 50 times. The DeLong test was used to assess discrimination between models(19). Calibration of the HF risk prediction model was plotted to obtain bias-corrected (overfitting-corrected) estimates of predicted vs. observed values based on subsetting the predictions into intervals. The prediction distribution of the models was plotted based on the order of the predicted risk for each patient.

4.4 Sensitivity analyses

Sensitivity analysis was computed for all patients from the TOPCAT study whose LVEF was 45-49%. These different models were developed for this population to predict all-cause mortality and HF hospitalization and were followed throughout the study period(20). The importance of each variable was calculated, and the incremental improvement in each variable was assessed over 50 cycles of simulation. In addition, 1-year all-cause mortality and HF hospitalization predictions were evaluated to see how the model's performance changed over a relatively short follow-up period.

REFERENCES

1. Pitt B, Pfeffer MA, Assmann SF, et al. Spironolactone for heart failure with preserved ejection fraction. N Engl J Med 2014;370:1383-92.

2. Pfeffer MA, Claggett B, Assmann SF, et al. Regional variation in patients and outcomes in the Treatment of Preserved Cardiac Function Heart Failure with an Aldosterone Antagonist (TOPCAT) trial. Circulation 2015;131:34-42.

3. Stekhoven DJ, Buhlmann P. MissForest--non-parametric missing value imputation for mixed-type data. Bioinformatics 2012;28:112-8.

4. Liaw A, Wiener M. Classification and regression by RandomForest. 2001.

5. Simon N, Friedman J, Hastie T, Tibshirani R. Regularization Paths for Cox's Proportional Hazards Model via Coordinate Descent. J Stat Softw 2011;39:1-13.

6. Freund Y, Schapire RE. A Decision-Theoretic generalization of On-Line learning and an application to boosting. J Comput Syst Sci 1997.

7. Bennett KP, Campbell C. Support Vector Machines: Hype or Hallelujah? Acm Sigkdd Explorations Newsletter 2000;2:1-13.

8. Breiman L, Friedman J, Olshen R, et al. Classification and regression tress. Encyclopedia of Ecology 1984;40:582-588.

9. Speiser JL, Miller ME, Tooze J, Ip E. A comparison of random forest variable selection methods for classification prediction modeling. Expert Syst Appl 2019;134:93-101.

10. Hapfelmeier A, Ulm K. A new variable selection approach using Random Forests. Comput Stat Data an 2013;60.

11. Ishwaran H, Lu M. Standard errors and confidence intervals for variable importance in random forest regression, classification, and survival. Stat Med 2019;38:558-582.

12. Segar MW, Vaduganathan M, Patel KV, et al. Machine learning to predict the risk of incident heart failure hospitalization among patients with diabetes: The WATCH-DM risk score. Diabetes Care 2019;42:2298-2306.

13. Hosmer DJ, Wang CY, Lin IC, Lemeshow S. A computer program for stepwise logistic regression using maximum likelihood estimation. Comput Programs Biomed 1978;8:121-34.

14. Zahrt AF, Athavale SV, Denmark SE. Quantitative Structure-Selectivity relationships in enantioselective catalysis: Past, present, and future. Chem Rev 2020;120:1620-1689.

15. Bazant, Lorenz. Linear regression and the LASSO. 2016.

16. Ogutu JO, Schulz-Streeck T, Piepho HP. Genomic selection using regularized linear regression models: Ridge regression, lasso, elastic net and their extensions. BMC Proc 2012;6 Suppl 2:S10.

17. Zou H, Hastie T. Zou H, Hastie T. Regularization and variable selection via the elastic net. J R Statist Soc B. 2005;67(2):301-20. Journal Of The Royal Statistical Society 2005;67:301-320.

18. Harrell FJ, Califf RM, Pryor DB, Lee KL, Rosati RA. Evaluating the yield of medical tests. JAMA 1982;247:2543-6.

19. Delong ER, Delong DM, Clarke-Pearson DL. Comparing the areas under two or more correlated receiver operating characteristic curves: A nonparametric approach. Biometrics 1988;44:837-45.

20. Angraal S, Mortazavi BJ, Gupta A, et al. Machine learning prediction of mortality and hospitalization in heart failure with preserved ejection fraction. JACC Heart Fail 2020;8:12-21.

Content	Page Number
**Online Table 1**. List of Candidate Variables included and excluded in the risk prediction analysis used for Predicting Mortality and Heart Failure Hospitalization in Patients with Heart Failure with mid-range Ejection Fraction.	5
**Online Table 2**. Variables in Rank Order of Importance for Predicting Heart Failure hospitalization over 1 Years Follow Up using VIMP metric.	9
**Online Table 3**. Variables in Rank Order of Importance for Predicting Heart Failure Hospitalization over Entire Study Period using VIMP metric.	10
**Online Figure 1**. Calibration plots of Lasso Cox regression Models for Predicting Mortality	11
**Online Figure 2**. Calibration plots of Random Forest Models for Predicting Heart Failure Hospitalization	11

Domain	Variable definition	Percent Missingness	Included/Excluded
Demographics	Age	0%	Included
Demographics	Gender	0%	Included
Demographics	RACE(Black/White/All Others)	0%	Included
Clinical history	Dyspnea: experienced in past year?	7.9%	Included
Clinical history	Orthopnea: experienced in past year?	5.0%	Included
Clinical history	Dyspnea on exertion: experienced in past year?	0.4%	Included
Clinical history	Rales: experienced in past year?	19.8%	Included
Clinical history	JVP: experienced in past year?	68.2%	Excluded
Clinical history	Edema: experienced in past year?	1.3%	Included
Clinical history	XRay: experienced in past year?	31.4%	Excluded
Clinical history	Previous hospitalization for CHF	0%	Included
Clinical history	Previous myocardial infarction	0%	Included
Clinical history	Stroke	0%	Included
Clinical history	Coronary artery bypass graft surgery	0%	Included
Clinical history	Percutaneous Coronary Revascularization	0%	Included
Clinical history	Angina Pectoris	0%	Included
Clinical history	Chronic Obstructive Pulmonary Disease	0%	Included
Clinical history	Asthma	0%	Included
Clinical history	Hypertension	0%	Included
Clinical history	Peripheral Arterial Disease	0%	Included
Clinical history	Dyslipidemia	0%	Included
Clinical history	Implanted cardioverter defibrillator	0%	Included
Clinical history	Pacemaker implanted	0%	Included
Clinical history	Atrial fibrillation	0%	Included
Clinical history	Atrial fibrillation: Paroxysmal atrial fibrillation	0%	Included
Clinical history	Atrial fibrillation: Chronic	0%	Included

	atrial fibrillation		
Clinical history	Thyroid disease	0%	Included
Clinical history	Thyroid disease: Hyperthyroidism	0%	Included but merged
Clinical history	Thyroid disease: Hypothyroidism	0%	Included but merged
Clinical history	Diabetes Mellitus (YES or NO or with Microvascular complications)	0%	Included
Clinical history	Duration since DM diagnosis (years)	0.2%	Included
Clinical history	Diabetes: Microvascular complications	0%	Included but merged
Clinical history	Microvascular complications: Retinopathy	0%	Included but merged
Clinical history	Microvascular complications: Nephropathy	0%	Included but merged
Clinical history	Microvascular complications: Neuropathy	0%	Included but merged
Social history	Does the subject currently smoke	0%	Included
Social history	Has subject ever been a smoker	0%	Included
Social history	How many Drinks do you consume per week (0/1-5/5-10/11+)	0%	Included
Social history	Activity Level (mets per week)	12.3%	Included
Social history	Does the subject currently live alone or with a spouse or significant other	0%	Included
Social history	Cooking Salt Score	0%	Included
Clinical history	Pulmonary	0%	Included
Clinical history	Cardiovascular	0%	Included
Clinical history	Neurological	2.5%	Included
Vitals	Body Mass Index	0.2%	Included
Vitals	Waist Circumference (cm)	1.2%	Included
Vitals	Heart rate	0%	Included
Vitals	Systolic blood pressure	0%	Included
Vitals	Diastolic blood pressure	0%	Included
Vitals	NYHA class 3&4 vs 1&2	0%	Included

Laboratory	Creatinine: Result (mg/dL)	0%	Included
Laboratory	Glomerular Filtration Rate	0%	Included
Laboratory	Sodium: Result (mmol/L)	0%	Included
Laboratory	Potassium: Result (mmol/L)	0%	Included
Laboratory	Chloride: Result (mmol/L)	4.8%	Included
Laboratory	CO2: Result (mmol/L)	59.7%	Excluded
Laboratory	Blood Urea Nitrogen: Result (mg/dL)	23.3%	Excluded
Laboratory	Glucose: Result (mg/dL)	0.4%	Included
Laboratory	WBC count: Result (k/uL)	0.8%	Included
Laboratory	Hemoglobin: Result (g/dL)	0.8%	Included
Laboratory	Hematocrit: Result (%)	1.0%	Included
Laboratory	Platelet Count: Result (k/uL)	1.5%	Included
Laboratory	Alanine Aminotransferase: Results (U/L)	0.6%	Included
Laboratory	Alkaline Phosphatase: Results (U/L)	3.3%	Included
Laboratory	Aspartate Aminotransferase: Results (U/L)	1.0%	Included
Laboratory	Total Bilirubin: Results (mg/dL)	1.0%	Included
Laboratory	Albumin: Results (g/dL)	3.1%	Included
Laboratory	Urine Microalbumin/Creatinine Ratio: Result (mg/g)	60.5%	Excluded
Laboratory	Brain natriuretic peptide (BNP) or N-terminal pro-BNP Result in (pg/ml)	59.5%	Excluded
Electrocardiographic	Ejection Fraction	0%	Excluded
Electrocardiographic	QRS Duration	0%	Included
Electrocardiographic	ECG_Atrial fibrillation/Flutter	17.9%	Included
Electrocardiographic	ECG_Bundle Branch Block - Yes/No indicator	17.9%	Included
Electrocardiographic	ECG_Ventricular paced rhythm	17.9%	Included
Electrocardiographic	ECG_Pathological Q waves	17.9%	Included
Electrocardiographic	ECG_Left ventricular	17.9%	Included

	hypertrophy		
Electrocardiographic	ECG_Other	17.9%	Included
Vitals	KCCQ: Physical Limitation score	1.0%	Included
Vitals	KCCQ: Symptom Stability score	0%	Included
Vitals	KCCQ: Symptom Frequency score	0%	Included
Vitals	KCCQ: Symptom Burden score	0%	Included
Vitals	KCCQ: Total Symptom score	0%	Included
Vitals	KCCQ: Self-Efficacy score	0%	Included
Vitals	KCCQ: Quality of Life score	0%	Included
Vitals	KCCQ: Social Limitation score	3.1%	Included
Vitals	KCCQ: Overall Summary score	0%	Included
Vitals	KCCQ: Clinical Summary score	0%	Included
Abbreviations: CHF, Cardiac Heart Failure; CVD, cardiovascular disease; ECG, electrocardiogram; JVP, Jugular venous pressure; KCCQ, Kansas City Cardiomyopathy Questionnaire; NYHA, New York Heart Association; WBC, white blood count;			

1_year hospitalization		
Variable	MeanDecreaseAccuracy	MeanDecreaseGini
HCT	5.43583711	1.45925840
KCCQ: Social Limitation score	4.68344908	0.75235815
KCCQ: Overall Summary score	3.94045644	0.68147952
KCCQ: Physical Limitation score	3.61124353	0.75279683
gfr	3.12051526	0.87594682
CABG	3.03608512	0.23193429
DM_DUR_YR	2.98172543	0.48940862
KCCQ: Symptom Frequency score	2.55675038	0.38300056
CR	2.44498486	1.19310453
ECG: Bundle Branch Block	2.40332832	0.08767703
KCCQ: Symptom Stability score	2.34896263	0.38300056
WBC	2.15165127	0.54260236
Glucose	2.13342905	0.76414541
KCCQ: Symptom Burden score	2.06582625	0.77182207
DBP	2.05378674	0.65555447
Hemoglobin	2.02574578	1.31446361
SBP	1.89013766	1.22706775
KCCQ: Total Symptom score	1.81014436	0.69986109
KCCQ: Clinical Summary score	1.77938632	0.70620665
PCI	1.76301413	0.15859814
Abbreviations:CABG, coronary artery bypass graft surgery; CR, creatinine; DBP, Diastolic blood pressure; DM_DUR_YR, Duration since Diabetes Mellitus diagnosis; ECG, electrocardiogram; gfr, glomerular filtration rate; HCT, Hematocrit; KCCQ, Kansas City Cardiomyopathy Questionnaire; PCI; percutaneous coronary intervention; SBP, Systolic blood pressure; WBC, white blood count;		

6_year hospitalization		
Variable	MeanDecreaseAccuracy	MeanDecreaseGini
KCCQ: Symptom Frequency score	6.37517686	2.09913912
KCCQ: Clinical Summary score	5.81667059	1.54551208
KCCQ: Overall Summary score	5.51602927	1.56823263
KCCQ: Social Limitation score	5.16515617	1.35152949
KCCQ: Physical Limitation score	4.92399841	1.31467458
KCCQ: Total Symptom score	4.62777670	1.56661541
Asthma	4.61574065	0.75760722
Race	4.56251122	1.01634137
KCCQ: Quality of Life score	4.47125623	1.07223577
DM_DUR_YR	4.39763179	1.59385957
gfr	4.02644573	1.85022165
THYROID	3.94666660	1.14764236
KCCQ: Symptom Burden score	3.77659581	0.90050249
SMOKE_EVER	3.69429342	0.77182207
AFIB	3.55732363	0.57600869
CR	3.44744758	2.02666868
SBP	2.93487079	1.98830043
BMI	2.83368495	2.71266322
Waist Circumference	2.64862170	2.28664637
Cooking Salt Score	2.40526750	1.18500801
Abbreviations: AFIB, Atrial fibrillation; BMI, body mass index; CR, creatinine; DM_DUR_YR, Duration since Diabetes Mellitus diagnosis; gfr, glomerular filtration rate; KCCQ, Kansas City Cardiomyopathy Questionnaire; SBP, Systolic blood pressure		

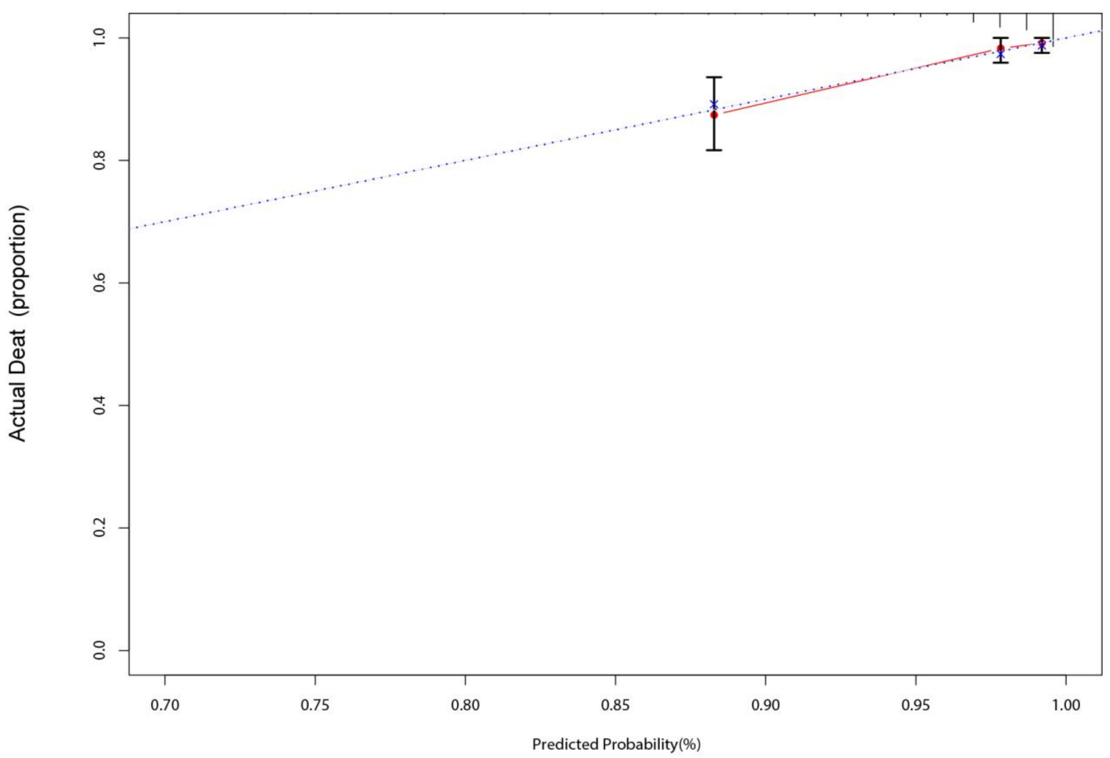
Actual Deat (proportion)
0.0
0.2
0.4
0.6
0.8
1.0
0.70
0.75
0.80
0.85
0.90
0.95
1.00
Predicted Probability(%)

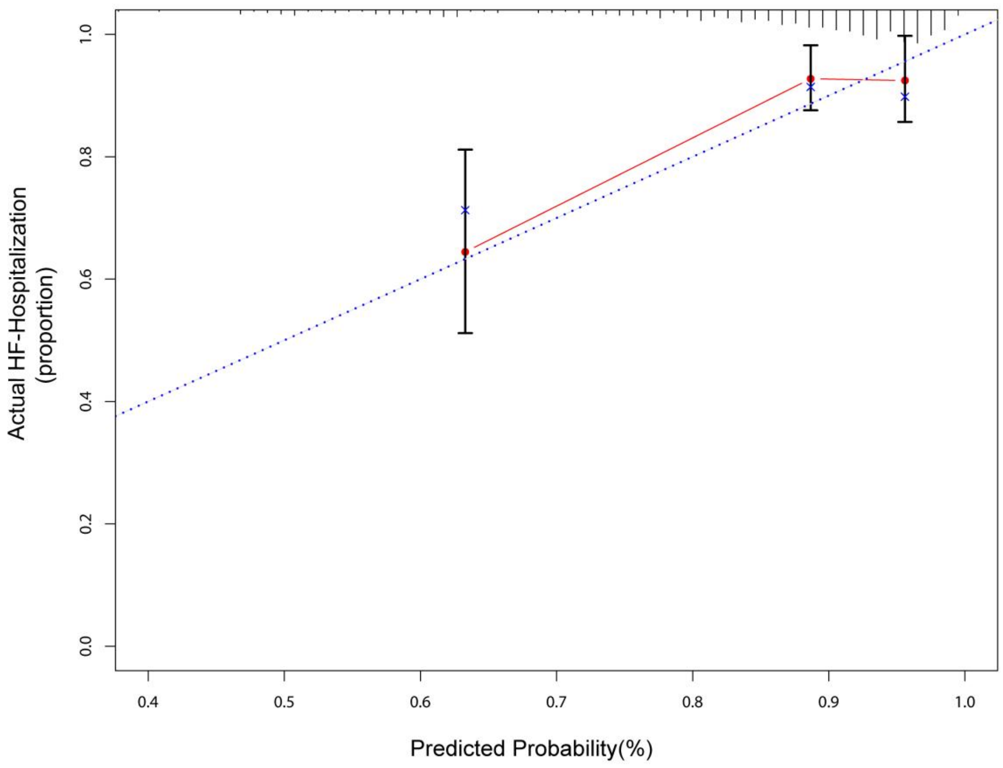
Actual HF-Hospitalization (proportion)
0.0
0.2
0.4
0.6
0.8
1.0
0.4
0.5
0.6
0.7
0.8
0.9
1.0
Predicted Probability(%)
